# Extramammary Paget's Disease Mimicking Squamous Cell Carcinoma In Situ: The Diagnostic Challenge of Recalcitrant Anogenital Lesions

**DOI:** 10.7759/cureus.113346

**Published:** 2026-07-25

**Authors:** Patricio Estrada Marentes, Paulina N Cortés López, Roberto Arenas, Elisa Vega-Memije

**Affiliations:** 1 Department of Dermatology, Hospital General Dr. Manuel Gea González, México City, MEX; 2 Department of Dermatology, Hospital General Dr. Manuel Gea González, Mexico City, MEX; 3 Department of Mycology, Hospital General Dr. Manuel Gea González, Mexico City, MEX

**Keywords:** anogenital lesions, cd44, ck7, dermatopathology, extramammary paget's disease, immunohistochemistry, mohs micrographic surgery, squamous cell carcinoma in situ

## Abstract

Extramammary Paget's disease (EMPD) is a rare intraepithelial adenocarcinoma that arises predominantly in apocrine gland-rich areas. Its clinical presentation is non-specific, frequently leading to erroneous clinical diagnoses, diagnostic delay, and prolonged morbidity. A 77-year-old female presented with a 10-year history of a dermatosis affecting the trunk, external genitalia, and the inner side of the right thigh, characterized by three erythematous-squamous plaques (1 to 4 cm in diameter). The patient had a previous histopathological diagnosis of squamous cell carcinoma in situ and was treated with topical 5% 5-fluorouracil for six months. Due to recalcitrance to therapy, a new incisional biopsy was performed on the thigh lesion. Histopathology revealed large, pleomorphic cells with clear cytoplasm and pagetoid migration. Immunohistochemistry showed positivity for CK7 and negativity for CD44, supporting the diagnosis of EMPD. The patient was subsequently referred for Mohs micrographic surgery. Taking new biopsies for recalcitrant lesions is critical. An appropriately selected, broad immunohistochemical panel is a fundamental tool to differentiate intraepidermal malignancies in complex anogenital cases.

## Introduction

Extramammary Paget's disease (EMPD) is a rare, slow-growing intraepithelial adenocarcinoma that is frequently misdiagnosed clinically [1]. It arises predominantly in apocrine gland-rich areas, such as the anogenital and axillary regions, and has a non-specific clinical presentation that often mimics benign inflammatory conditions like eczema or candidiasis [2]. Furthermore, histopathologically, it can be easily confused with other intraepidermal malignancies, most notably squamous cell carcinoma in situ (SCCIS) and melanoma in situ (MIS), especially when clear cell changes and pagetoid spread are present [3].

In this article, we present the case of a patient with a recalcitrant anogenital lesion to highlight the diagnostic challenge of differentiating EMPD from its morphological mimics. We emphasize the necessity of clinicopathological correlation and an appropriately selected immunohistochemical panel in correcting previous misdiagnoses and guiding surgical management.

This article was previously presented as a poster at the Dermajal 2026 Congress.

## Case presentation

**Figure 1:**
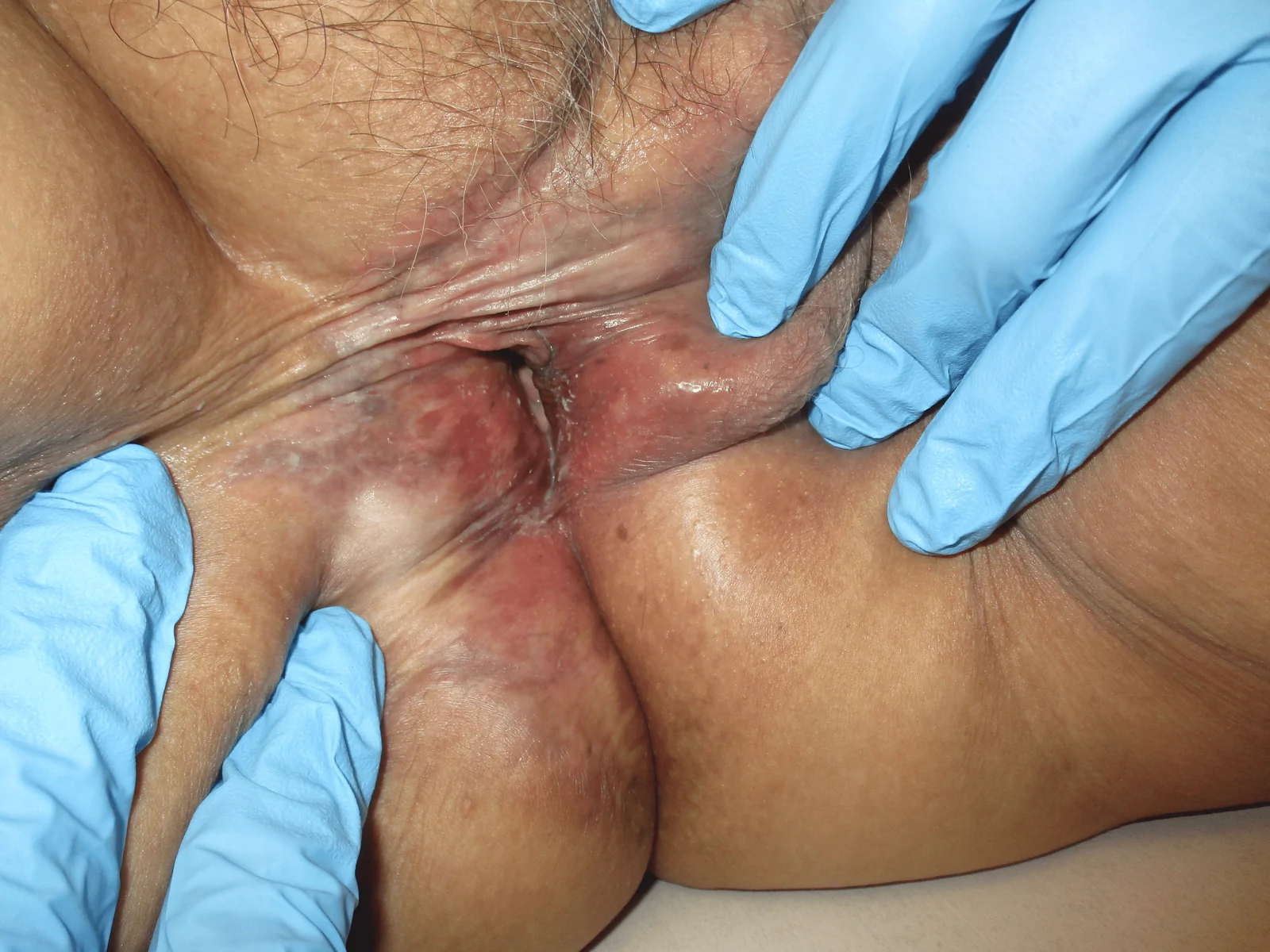
Clinical presentation of the anogenital lesions Erythematous-squamous plaques with irregular but well-defined borders and central exulceration, affecting the external genitalia and the inner aspect of the right thigh.

A 77-year-old female presented with a dermatosis of 10 years' evolution, localized on the trunk and affecting the external genitalia and the inner aspect of the right thigh. Physical examination revealed three erythematous-squamous plaques, ranging from 1 cm to 4 cm in diameter. The lesions exhibited an ovoid shape with irregular but well-defined borders and central exulceration on the surface (Figure 1). All three anatomically separated lesions were clinically considered manifestations of the same process. 

The patient provided a previous written histopathological report with a diagnosis of squamous cell carcinoma in situ (SCCIS); however, the original slides were unavailable for our review, and no immunohistochemistry had been performed during that initial assessment. Based on this report, treatment with topical 5% 5-fluorouracil every 24 hours was initiated and maintained for six months.

**Figure 2:**
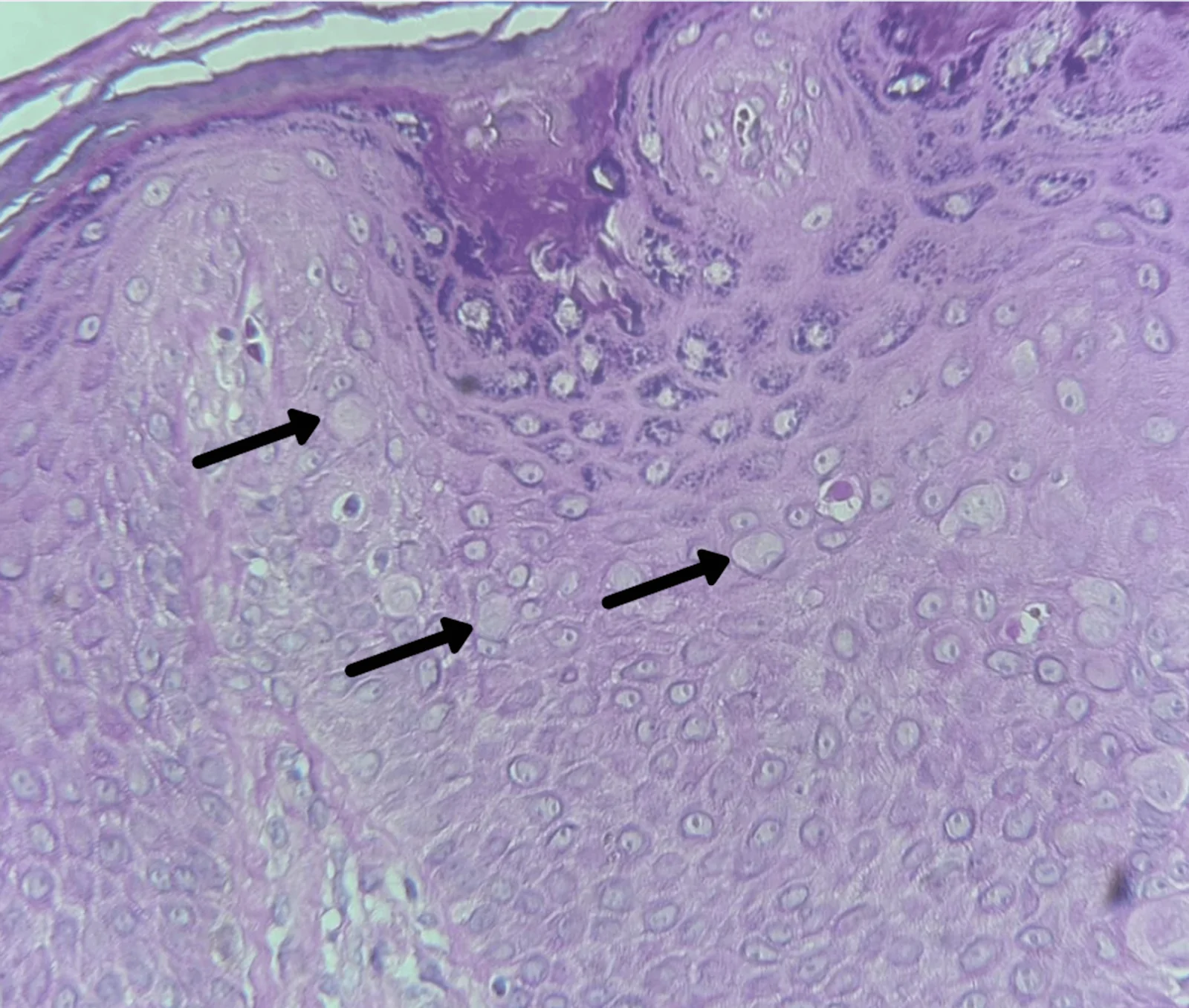
Histopathological features of the lesion (H&E stain) Hyperkeratotic, compact lamellar stratum corneum with hypergranulosis. The epidermis shows irregular acanthosis with scattered, large pleomorphic cells featuring prominent nuclei and clear cytoplasm (arrows) (H&E stain, original magnification: 40x).

Given the torpid evolution and the persistence of the lesions despite the targeted therapeutic regimen, it was decided to perform a new incisional biopsy on the most representative 4 cm plaque on the right inner thigh. The new histopathological study revealed a hyperkeratotic, compact lamellar stratum corneum with hypergranulosis, overlying an epidermis with irregular acanthosis and elongation of the rete ridges. Large, scattered, pleomorphic cells with prominent nuclei and clear cytoplasm were observed (Figure 2). No dermal invasion, adnexal involvement, or lymphovascular invasion was identified in the evaluated sections.

**Figure 3:**
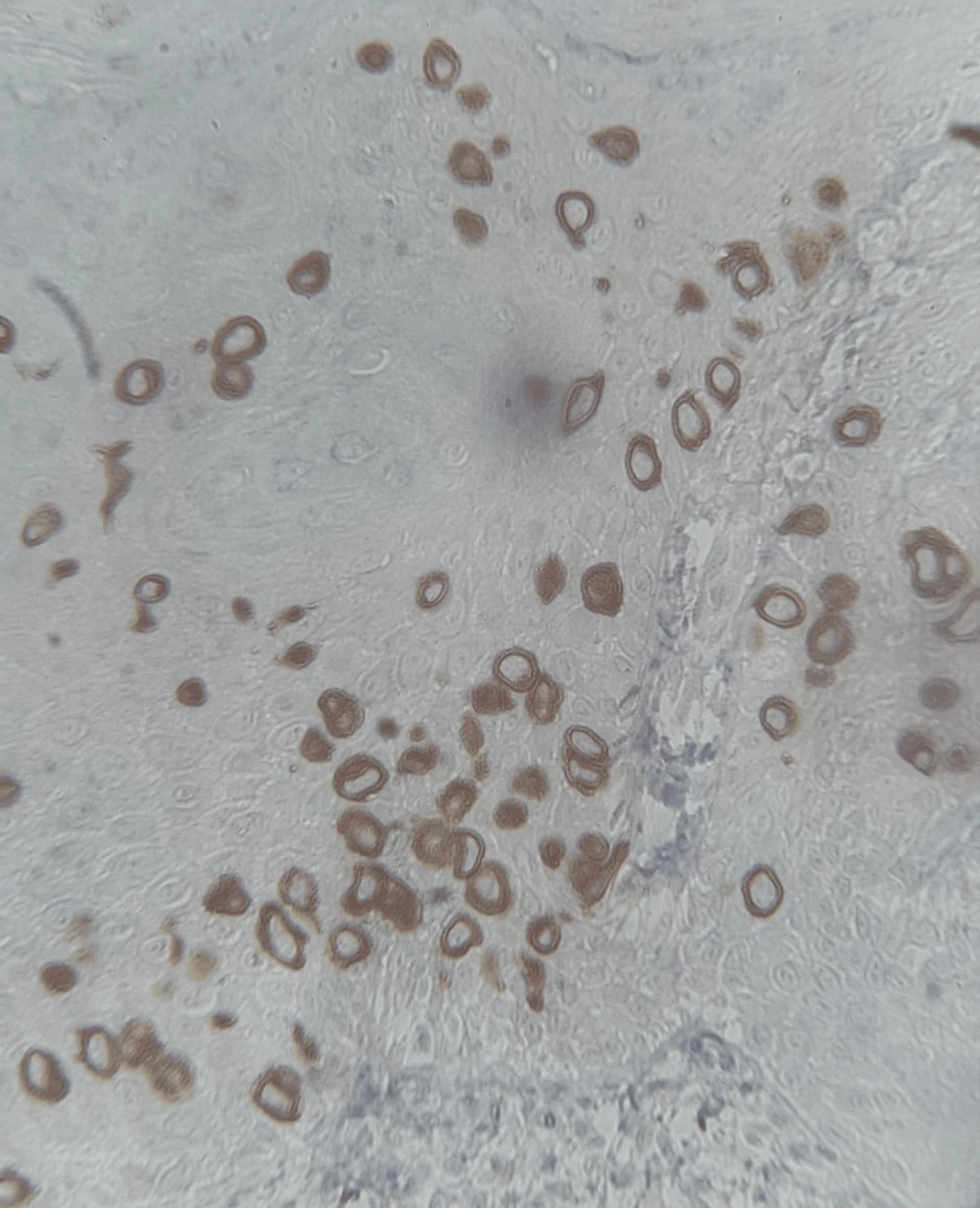
Immunohistochemistry for CK7 The large, pleomorphic pagetoid cells demonstrate strong positive cytoplasmic staining for CK7 (Chromogen: DAB, original magnification: 40x).

Given the presence of these pleomorphic cells exhibiting pagetoid migration, an immunohistochemical evaluation was requested. The atypical cells stained positive for CK7 and negative for CD44 (Figure 3). Together with the clinical presentation, these findings supported the diagnosis of extramammary Paget's disease. Following the diagnosis, the patient was referred to an external surgical center for Mohs micrographic surgery (MMS) evaluation.

## Discussion

Extramammary Paget's disease (EMPD) is a rare cutaneous malignancy that poses a significant diagnostic challenge. The mean time from symptom onset to diagnosis is approximately 36 months, though our patient experienced a remarkably prolonged 10-year evolution [1].

In this case, the initial misdiagnosis highlights the morphological overlap between these entities. On standard hematoxylin and eosin (H&E) staining, EMPD, SCCIS, and melanoma in situ (MIS) can all demonstrate a pagetoid scatter of large, atypical cells with clear cytoplasm throughout the epidermis [3]. Because SCCIS can exhibit clear cell changes that closely mimic the classic appearance of Paget cells, the differential diagnosis is exceedingly difficult based on morphology alone [3].

Immunohistochemistry is therefore an essential tool to resolve this diagnostic dilemma and differentiate these three close mimics. MIS is typically positive for melanocytic markers such as S100, SOX10, or Melan-A, and negative for CK7. Once MIS is ruled out, distinguishing EMPD from SCCIS becomes the primary challenge. CK7 is a highly sensitive marker for EMPD, demonstrating positivity in 93% to 100% of cases; however, because a subset of pagetoid SCCIS can unexpectedly express CK7, a broader panel approach is generally necessary [4]. While markers such as p63 are classically used to confirm squamous origin in SCCIS and are absent in EMPD [4], our evaluation utilized CD44, which is typically expressed in atypical keratinocytes of SCCIS and lost in EMPD. The combination of CK7 positivity and CD44 negativity in our patient provided crucial support for the diagnosis of EMPD, overriding the initial morphology-based report.

The clinical evolution of the patient further correlated with the final diagnosis. Topical 5-fluorouracil demonstrates high efficacy for SCCIS, with complete clearance rates exceeding 90% in primary therapy [5]. Conversely, it lacks adequate evidence for primary EMPD and is not considered a curative standard [6]. While treatment failure is not diagnostically specific and could reflect adherence issues or lesion extent, in this context, the persistent lesions after six months of continuous therapy raised the clinical suspicion of an incorrect initial diagnosis.

Because MMS achieves significantly lower recurrence rates for anogenital EMPD (7-12%) compared to wide local excision (26-37%), the patient was referred for this surgical modality [7]. The layer-by-layer assessment provided by MMS is critical for EMPD, as the disease is characterized by ill-defined clinical borders and extensive subclinical spread [1,7].

## Conclusions

Extramammary Paget's disease can closely mimic squamous cell carcinoma in situ and melanoma in situ, both clinically and histopathologically. This case underscores the critical importance of maintaining a high index of suspicion and performing repeat biopsies when recalcitrant lesions fail to respond to standard therapies, regardless of previous histopathological reports. To accurately differentiate these intraepidermal malignancies, strict clinicopathological correlation and the application of an appropriately selected, broad immunohistochemical panel are essential to prevent prolonged morbidity and guide definitive surgical management.
